# Easypod^TM^ adherence in children with growth disorders treated with r-hGH. A preliminary Italian experience

**DOI:** 10.1186/1687-9856-2013-S1-P54

**Published:** 2013-10-03

**Authors:** Sandro Loche, Piernicola Garofalo, Mariacarolina Salerno, Mohamed Maghnie, Maria Rosaria Licenziati, Giuliana Marcella Cardinale, Giuseppe Citro, Manuela Caruso Nicoletti, Salvatore Longobardi, Raffaella Perrone

**Affiliations:** 1 Ospedale Microcitemico, Cagliari, Italy; 2 Ospedale “Villa Sofia-Cervello”, Palermo, Italy; 3 Università “Federico II”, Napoli, Italy; 4 Istituto G. Gaslini, Genova, Italy; 5 AORN Santobono – Pausillipon, Napoli, Italy; 6 Presidio Ospedaliero “F. Ferrari” Casarano, Lecce, Italy; 7 Polo Sanitario “Madre Teresa di Calcutta”, Potenza, Italy; 8 Policlinico Universitario, Catania, Italy; 9 Merck Serono S.p.A., Roma, Italy

## 

Recombinant human growth hormone (r-hGH) is indicated for pediatric patients with a variety of growth disorders. Treatment with r-hGH requires long-term daily injections (1). Non-adherence or low adherence to treatment is associated with less favorable clinical outcomes, lower quality of life and higher healthcare costs (2,3). easypod^TM^ has been specifically designed to improve patients’ ease of daily use and to minimize discomfort and pain (4,5).

Aim of this observational, national and multicentre study was to monitor the adherence to r-hGH treatment using easypod^TM^, which records time, date and dose of injections in patients with growth disorders during the 1st year of treatment.

98 pre-pubertal short stature patients with growth disorders (60 male and 37 female, 1 screening failure, median age 10 years) were enrolled in the study. So far data are available for 54 subjects. All subjects underwent a baseline visit, and then after 6 and 12 months. 8 patients were withdrawn from the study prematurely.

Data regarding r-hGH administration was uploaded from the autoinjectors. Auxological parameters, routine blood chemistry and hematology values were collected.

Adherence during the 1st year has been calculated. Adherence was defined as the proportion of injections correctly administered out of expected total number of injections during the observational period. A patient was considered as adherent if he/she correctly administered >92% of expected total number of injections during the observation period. Data on the 54 available patients show that 25 patients (46.3%) were adherent to treatment while 29 patients (53.7%) were not. The impact of low adherence on clinical and biochemical parameters is under evaluation.

The study will run until January 2013. Data obtained from this study will allow to explore the potential impact of adherence to r-hGH on growth outcomes after 1st year of treatment. The long-term adherence will be evaluated in the easypod^TM^ connect observational study, also ongoing in Italy.

